# Conservative Custom Wire and Loop-Assisted Retrieval of a Long Separated Instrument Extending Beyond the Apex: A Case Report

**DOI:** 10.7759/cureus.115469

**Published:** 2026-08-30

**Authors:** Maria Thomas, Praveena G, Anjana Ashok, Anulakshmi T P

**Affiliations:** 1 Conservative Dentistry and Endodontics, Azeezia College of Dental Sciences and Research, Kollam, IND

**Keywords:** instrument retrieval, masserann kit, root canal treatment, separated instrument, ultrasonics, wire and loop technique

## Abstract

Separation of endodontic instruments is a recognized procedural complication that can hinder effective canal debridement and compromise treatment outcomes, making successful retrieval essential whenever feasible. A 30-year-old female presented with pain in the upper right posterior region associated with tooth 16, diagnosed as asymptomatic irreversible pulpitis with symptomatic apical periodontitis. During root canal treatment, a rotary NiTi file fractured in the mesiobuccal canal and extended beyond the apex. Owing to favorable visibility and fragment length, retrieval was attempted using a conservative combination approach involving ultrasonics for fragment exposure, Masserann trephine troughing, and a custom-made syringe-based wire loop technique. The separated instrument was successfully retrieved, after which canal preparation, obturation, and restoration were completed. This case highlights that the combined use of ultrasonics, the Masserann technique, and a custom wire loop can provide an effective, minimally invasive, and cost-effective method for retrieval of separated instruments in suitable cases.

## Introduction

Separation of an endodontic instrument within the root canal is a significant procedural complication that may adversely affect the outcome of root canal treatment. A separated instrument can obstruct access to the apical portion of the canal, thereby interfering with adequate cleaning, shaping, and disinfection. Instrument separation is influenced by several factors, including root canal curvature, instrument design and manufacturing defects, dynamics of instrument use, instrumentation techniques, and operator skill [1].

Management of a separated instrument generally involves three approaches: removal of the fragment, bypassing it to regain access to the apical portion of the canal, or cleaning and obturating the canal up to the level of the obstruction when further retrieval or bypass is not feasible [2]. The decision to attempt retrieval is influenced by factors such as the location and length of the fragment, canal anatomy and curvature, and the type of fractured instrument. Retrieval techniques include ultrasonic methods, trephine bur techniques, microtube and loop systems, laser-assisted retrieval, and commercially available instrument removal systems [3]. However, some retrieval techniques may require removal of surrounding dentin to gain access to and loosen the fragment, which may increase the risk of weakening the root.

Instrument fracture primarily occurs through cyclic fatigue or torsional stress. Cyclic fatigue results from repeated tension-compression cycles during instrumentation in curved canals, whereas torsional fracture occurs when an instrument becomes locked within the canal while rotational forces continue to act on the instrument [4]. Understanding these mechanisms is important because the location and condition of the separated fragment can influence the difficulty and approach to its retrieval.

The wire loop technique is a minimally invasive retrieval method in which a loop formed from a thin wire is maneuvered around the separated fragment and tightened to facilitate its removal. The Masserann technique uses trephine burs to selectively remove dentin surrounding the fragment, allowing it to be exposed and loosened before retrieval with specialized extractors. Piezoelectric ultrasonic tips can also be used to conservatively remove dentin and generate vibrations that aid in loosening the separated fragment [3]. Combining these techniques may provide complementary advantages: ultrasonics can facilitate conservative exposure and loosening of the fragment, the Masserann technique can provide controlled access when necessary, and a customized wire loop can engage and retrieve the fragment without relying solely on specialized extraction devices.

This case report describes the successful retrieval of a long separated instrument extending beyond the apex using a combination of piezoelectric ultrasonics, the Masserann technique, and a custom-made wire and loop method, demonstrating a conservative and adaptable approach for the management of a challenging separated instrument.

## Case presentation

A 30-year-old female patient reported to the department with a chief complaint of pain in the upper right posterior tooth region for the past two years. The patient gave a history of severe nocturnal pain one week prior, for which she had taken medication from a local dental clinic. She also reported a history of dental caries in the same tooth for two years, associated with sensitivity to hot and cold stimuli.

On clinical examination, a deep Class II carious lesion was observed in relation to tooth 16, which was tender on percussion. Radiographic evaluation revealed coronal radiolucency involving enamel, dentin, and pulp, along with periapical widening and loss of lamina dura in the mesiobuccal root. Pulp sensibility tests showed a delayed response to both electric pulp testing and cold thermal testing (lingering response for 5 seconds) when compared to the control tooth.

Based on the clinical and radiographic findings, a diagnosis of symptomatic irreversible pulpitis with symptomatic apical periodontitis was established. The treatment plan consisted of root canal treatment followed by full-coverage crown restoration in relation to tooth 16.

The tooth was anesthetized using 2% lignocaine hydrochloride containing 1:100,000 epinephrine. An endodontic access cavity was prepared, and the canals (MB1, MB2, DB, and P) were located using a DG 16 explorer. Pulp extirpation was carried out using size 6, 8, and 10 K-files. Coronal preflaring of the mesiobuccal, distobuccal, and palatal canals was performed using an orifice enlarger (Hanudent TopFlex SX), followed by working length determination.

During biomechanical preparation, a rotary NiTi file (Hanudent TopFlex 15/.04) fractured in the mesiobuccal canal. The fractured segment (11 mm) was visible at the canal orifice level (Figure 1a). Radiographic examination confirmed the presence of the separated instrument extending beyond the apex (Figure 1b). Considering the length and coronal accessibility of the fragment, retrieval was planned using a combination of the Masserann technique and a custom-made wire and loop method.

A custom wire loop device was fabricated chairside using a disposable syringe and stainless steel wire. The conventional needle was replaced with a wider gauge needle to facilitate passage of the wire. A 0.05 mm diameter stainless steel orthodontic wire was inserted through the needle lumen, and both free ends were retrieved from the proximal end, thereby forming a loop at the distal tip. The syringe plunger was removed, and shallow grooves were prepared on its surface using a diamond bur to secure the wire. The free ends of the wire were passed through the syringe barrel and positioned along the grooves on the plunger. The plunger was reinserted, ensuring stabilization of the wire, and the protruding ends were tightened by twisting. The loop at the needle tip functioned as a lasso that could be maneuvered around the exposed fragment. Retraction of the plunger resulted in tightening of the loop, enabling firm engagement of the fractured instrument (Figure 1c).

**Figure 1:**
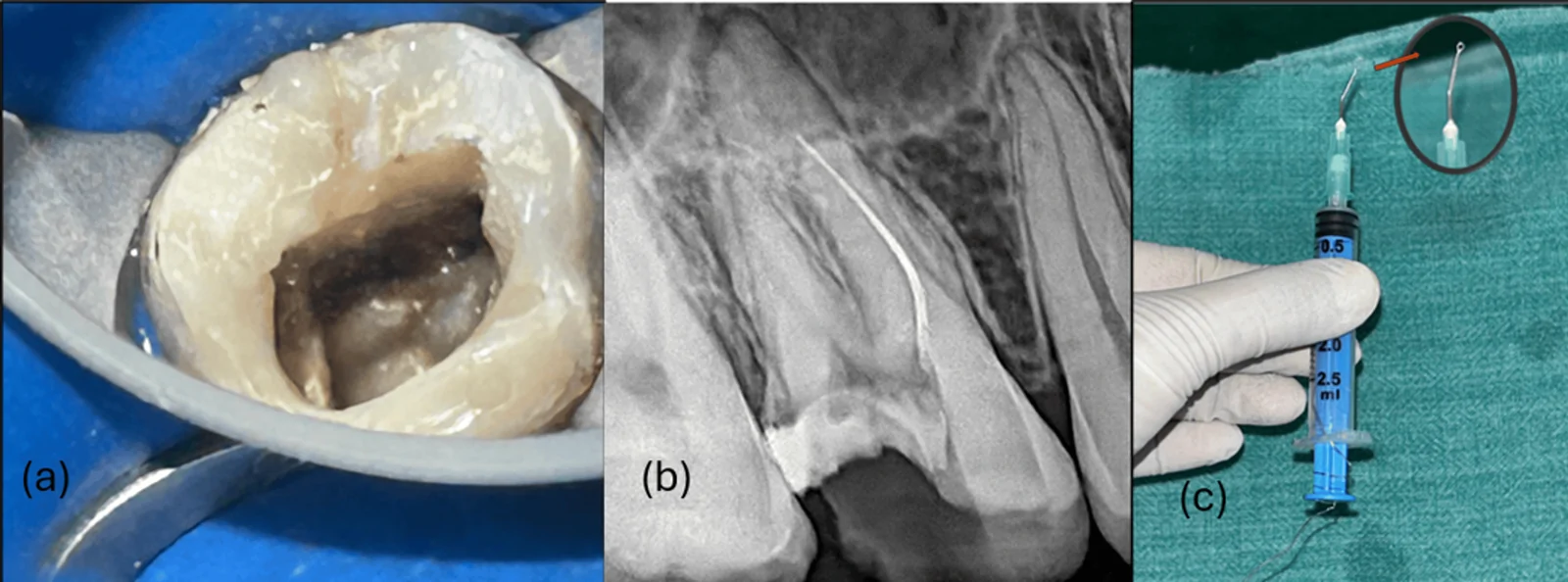
(a) Instrument visible at canal orifice. (b) Radiograph showing separated instrument extending beyond apex. (c) Custom-made wire-loop device.

The loop diameter was maintained at approximately 0.8-1.0 mm to correspond with the estimated coronal diameter of the fractured instrument. A pre-bend of 30-45° was incorporated near the needle tip to facilitate alignment with the mesiobuccal canal anatomy. Additionally, the loop was designed in an elongated configuration to enhance adaptation along the long axis of the fragment.

At the subsequent visit, under rubber dam isolation, pre-endodontic buildup was performed using composite resin. Under magnification, the access cavity was refined to achieve improved visibility and straight-line access. The fractured instrument was visualized, and a communication between MB1 and MB2 canals was noted. An ultrasonic tip (E4) was used to expose approximately 4 mm of the coronal portion of the fragment (Figure 2a).

A preselected trephine bur from the Masserann kit was then used in an anticlockwise direction to create a circumferential trough around the coronal end of the fragment by selective dentin removal. An extractor was subsequently attempted; however, the fragment remained tightly engaged within the canal (Figure 2b).

Further loosening was achieved by rotating a DG 16 explorer counterclockwise under minimal apical pressure to create a groove around the coronal end of the fragment. Simultaneously, low-intensity ultrasonic energy was applied indirectly to the DG 16 explorer using a universal scaler tip (indirect ultrasonic activation), which aided in creating additional space and loosening the fragment.

The custom-made wire loop was then introduced into the canal and maneuvered around the exposed fragment. Once a firm grip was achieved, as confirmed by tactile feedback, the wire was gently withdrawn along with the syringe plunger, resulting in successful retrieval of the fractured instrument (Figure 2c, 2d). Radiographic confirmation of complete removal was obtained (Figure 2e).

**Figure 2:**
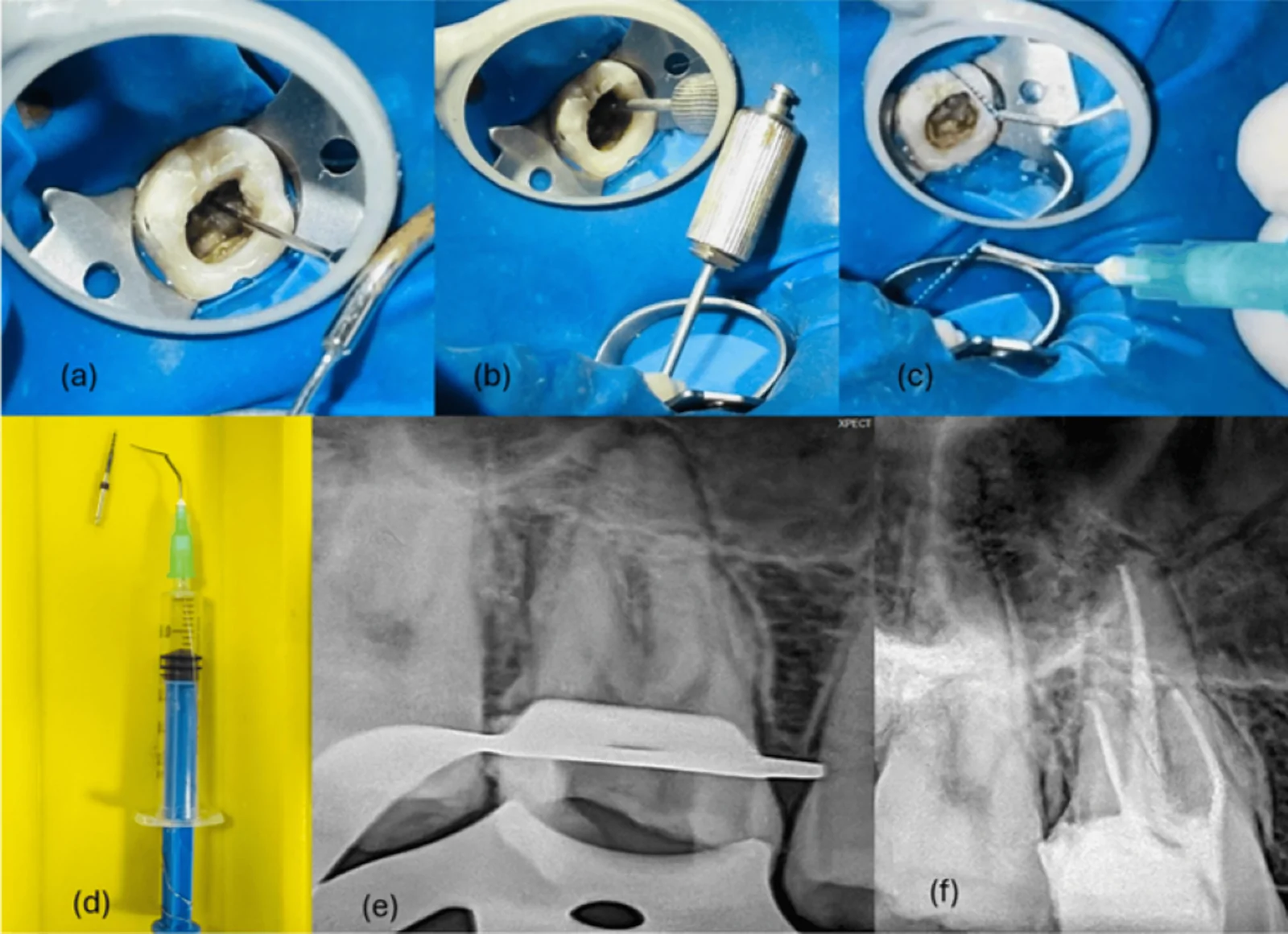
(a) Ultrasonic exposure of the coronal portion of the fractured instrument. (b) Dentin troughing around the fragment using a Masserann trephine. (c) Retrieval of the separated instrument using a custom-made wire loop technique. (d) Separated instrument within loop. (e) Radiograph after instrument retrieval. (f) Radiograph after obturation.

Subsequently, the canals were prepared and enlarged up to size 30/.04, followed by obturation using gutta-percha and zinc oxide eugenol sealer (Figure 2f).

## Discussion

The management of a separated instrument (SI) in the root canal requires careful clinical judgment. The decision to remove, bypass, or retain the fragment should be based on a thorough evaluation of the potential benefits and risks. Although the presence of an SI does not necessarily result in treatment failure, it may hinder adequate cleaning and disinfection of the root canal system, thereby compromising long-term prognosis [3]. At the same time, attempts at retrieval may lead to procedural complications such as ledge formation, perforation, canal transportation, or excessive removal of dentin [5]. Therefore, the clinician must carefully weigh the likelihood of successful retrieval against the risk of iatrogenic damage.

Among the available techniques, the Masserann kit (Micro-Mega, Besançon, France) is a commonly used system for instrument retrieval. It consists of hollow trephine burs and extractor tubes designed to engage and remove the fractured fragment. The trephine burs are operated in a counterclockwise direction to create a circumferential trough around the coronal portion of the fragment, allowing engagement by the extractor. However, due to the relatively large diameter of the trephines and extractors (1.1-2.4 mm), significant dentin removal is often required, which may weaken the root structure and increase the risk of perforation or vertical root fracture [6,7].

In the present case, successful retrieval was achieved through a conservative and customized approach using a wire loop technique. The effectiveness of this method was enhanced by tailoring the loop design according to the dimensions and location of the fractured fragment. The loop diameter was maintained at approximately 0.8-1.0 mm to correspond with the coronal diameter of the fractured size 15/.04 instrument, enabling effective encirclement and engagement. A pre-bend of 30-45° near the needle tip facilitated alignment with the mesiobuccal canal anatomy, improving accessibility. Furthermore, the elongated configuration of the loop allowed better adaptation along the long axis of the fragment, thereby enhancing mechanical grip and enabling controlled retrieval. These modifications minimized unnecessary dentin removal and reduced the risk of apical displacement of the fragment.

Nickel-titanium (NiTi) rotary instruments are generally more challenging to retrieve than stainless steel (SS) instruments due to their tendency to bind within canal walls as a result of rotational motion. Additionally, NiTi instruments are more susceptible to secondary fracture during retrieval procedures, particularly when ultrasonics are used [8]. In curved canals, fractured NiTi fragments tend to lie against the outer canal wall due to their flexibility, further complicating retrieval [8]. It has also been reported that longer fragments are generally easier to retrieve due to better accessibility of their coronal portion [3]. In the present case, the fractured segment was relatively long and partially visible, which contributed to the favorable outcome.

The use of ultrasonics in combination with magnification, such as a dental operating microscope, is widely regarded as an effective method for instrument retrieval. However, improper use of ultrasonics may generate heat, leading to further fracture of the instrument or damage to the surrounding dentin [9]. Excessive dentin removal during ultrasonic application may also result in procedural errors such as perforation or ledge formation. In this case, indirect ultrasonic activation was employed to minimize these risks while facilitating loosening of the fragment.

Additionally, favorable canal anatomy played a crucial role in the success of retrieval. The relatively straight and wide canals in this case allowed better access and visibility, enabling a more conservative approach. The use of trephine burs aided in creating limited space around the fragment, while the custom-made wire loop technique provided a secure and controlled method for retrieval. The combination of these techniques resulted in successful removal of the separated instrument with minimal loss of tooth structure.

## Conclusions

Management of separated instruments remains a clinical challenge in endodontic practice. This case demonstrated that successful retrieval of a long nickel-titanium instrument extending beyond the apex can be achieved through a conservative combination of ultrasonic exposure, selective dentin troughing with the Masserann technique, and a customized wire-loop retrieval method. The customized wire-loop device provided effective engagement of the fragment while minimizing unnecessary dentin removal in this case. Careful case selection, adequate visualization, and preservation of tooth structure were key factors contributing to the favorable outcome. The described technique may serve as a simple, economical, and minimally invasive alternative for retrieval of separated instruments in appropriately selected cases.
